# Diseases of the Mouth

**Published:** 1911-05

**Authors:** Robin Adair

**Affiliations:** Professor Oral Prophylactic and Alveolar Pyorrhea, Southern Dental College, Atlanta, Ga.


### DISEASES OF THE MOUTH.

By Robin Adair, M.D., D.D.S.

*Professor Oral Prophylactic and Alveolar Pyorrhea, Southern Dental College, Atlanta, Ga.*

Diseases of the mouth are due more consideration in medical treatment. Text books barely mention the names, and not being stressed in the colleges, is responsible for the almost universal neglect of this field. This neglect so often results in loss of masticating surface, part of a jaw or even death of the patient.

The first I mention of these diseases is neoplasm. The physician is accustomed to seeing large growths and severe symptoms in other parts of the body and overlooks what the patient calls attention to in the mouth as the little "bump" or "ulcer." The surgical record of any hospital gives a long list of horrible operations and many deaths that might have been prevented by immediate attention to all growths that may have been suspicious.

The cause of these growths is often of dental origin, bad bridge work, sharp edges of teeth or tartar—certainly I urge that the dentist should be consulted, but they are not prepared to make correct diagnosis, for they cannot make a blood count or a microscopical section. The custom of treating such cases with irritating drugs is to be condemned, as it only adds fuel to the fire. If indicated, prompt removal should be made at their inception, as delay in this region is more dangerous than in other parts of the body.

### Diseased Gums.

The next class of diseases which I specially call your attention to are those of the Alveolar process and gums. Symptoms range from single gingivitis when the gums are red and bleed at the slightest touch, to the advanced stage where there is a constant discharge of pus with destruction of tissue and impairment of health. *Names* and *Definitions* range everywhere from Riggs disease, Pyorrhea, Alveolaris, Infectious Alveoletis, Cemento-perostitis and calcic inflammation, Blenorrhea Alveolaris, Hemotoginic-pericementitis, Phagademic Pericementitis, Interstitial gingivitis, Diabetic Pyorrhea and many others too numerous to mention, all describing some phase of the same condition.

Causes.—The causes are given as both constitutional and local—several under the head of constitutional are—general condition of health, hereditary constitutional diseases, excessive lime salt secretions, uric acid states, toxic agents introduced into the system. The local causes are germs, meat eating, lack of proper attention to the mouth during sickness, but the greatest responsibility lies under the head of uncleanliness.

*Pathology.*—The alveolar process is not true bone, but a transitory structure. Its only purpose is to support the teeth, and when they are lost, its function and use is over and it is absorbed. The alveolar process is also an end organ, in that numerous blood vessels end in its substance. Having no free circulation, all poisons quickly accumulate in its substance and congestion follows. On the other hand, outside irritants as tartar, sordes, etc., cause a congestion with resultant absorption and exfoliation of the teeth. The early stages present a very red, inflamed gum margin, which bleeds at the slightest touch. The appearance of the next stage is more difficult for the physician to diagnose. The redness may have disappeared, but the gums have a puffed appearance and do not cling to the teeth. A probe can easily be passed to considerable depth between the teeth and gum. The latter stage is absorption of process, recession of gums and loose teeth.

*Symptoms.*—The first symptom noticed by patient is bleeding of gums when brushing the teeth. There is rarely any pain at any stage of the disease, and its development is so gradual that the patient is surprised when told of the condition. From the incipiency to the last stage may be passed without the patient being aware of any trouble. Length of development may extend over a period of ten years—children as well as adults are susceptible.

*Complications.*—Complications are numerous and more severe and serious than the disease itself, with the result that the physician more often treats the symptom instead of getting at the real cause. Just a few cases to illustrate this: Mr. ——— a wealthy Jew of about fifty years of age, referred to me by a surgeon who had recognized Pyorrhea Alveolaris as the cause of his run-down condition and extreme nervousness, indigestion and indicanuria. I made a date for operation which patient afterwards cancelled, giving his reason that a general practitioner of medicine had convinced him that his condition would not admit of the operation, and that a trip to Europe was the one thing for him to do at this time. I wonder if this doctor evacuates all his abscesses and removes necrotic tissue by trips to Europe. Not a bit of it, and he should have advised differently here for the man's mouth was a mass of necrotic tissue with a discharge of pus into it of about one ounce per day, which he had swallowed each day, poisoning the system; and yet a simple operation which would have brought immediate results like unto removing a splinter from a suppurating finger.

Case A. ————— a wealthy lady of large and corpulent statue under care of physician, who was treating her for kidney trouble—no examination or treatment was advised for her mouth. She came to me for an aching molar. On examination, I found one of the worst infected mouths I ever beheld. Decomposed bread particles packed in pockets between teeth—gums flabby and greatly congested. One week after treatment the indican had about disappeared and her general health greatly benefitted.

Case B. ————— patient had entered into a contract (two months) with a stomach specialist. No examination was made of her mouth. Soon after arriving in the city she came with a friend who had an engagement at my office. Out of curiosity I suppose, she asked for an examination of her mouth to see if any fillings were necessary. The mouth presented a bad case of Pyorrhea Alveolaris, and without knowing that she was under treatment, I described to her the complications which might happen and the benefit of an operation. She took my advice and improved so rapidly that she returned home in three weeks and has been well ever since. Needless to say, the stomach man got the credit for the wonderful and speedy cure—when the truth was that he would have been treating her for two months if her mouth had not been attended to.

*Diagnosis.*—The physician probably sees more of these cases than the dentist, in that the general symptoms are those for which the patient seek relief and treatment—the physician looks into the mouth, but the tip of the tongue is not all he should examine. My earnest advice and the purpose of this paper is to urge you to extend your field of vision to the gums. A little endeavor in this direction, and a few consultations with a dentist will soon give you a knowledge of the diagnostic points. The diagnosis is made from the symptoms and Pathological Anatomy above described.

*Treatment.*—The local surgical treatment is the work for the dentist. In this paper, I refer only to that period in which the mouth of the patient is under the care of the physician, who should refer the patient to the dentist as soon as practicable. Says Dr. Agnes de Lima of the Bureau of Municipal Research of New York. Doctors still prescribe tonics for invalids whose diseased mouths are drawing their vitality more than any other cause. Fortunes are spent to attempt to cure tubercular patients, who reinfect themselves every time food, medicine and saliva pass over their diseased cavities and gums. Millions are spent in purifying the water supply and the soils. Medical Institutions are endowed to stamp out the contamination of food and air by 'pathogenic bacteria,' but the prime breeding place for germs (the human mouth) is uncared for.

Many of the so-called throat, eye, stomach, intestinal liver and kidney troubles are due to a diseased mouth. And all the medicine and medical treatment in Christendom cannot cure them. They must have attention to the mouth if you expect them to be restored to health. The worst part of the picture is so many mouths which were in a normal condition before their illness soon after convalescence develop diseased gums, just because the physician had not given the proper attention to this organ while it was under his care. In this connection, I wish to quote from a paper I read recently before the Fulton County Medical Society on "The Care of the Teeth During Sickness." A sick patient seems to lose sight of what little care he formerly gave his mouth. He loses the cleansing advantages incident to vigorous chewing, drinking, of exercise and areation of the mouth, with the result that it soon becomes a hotbed of filth and disease, making a human culture tube, with all conditions of heat, moisture and food—it would be impossible for the bacteriologist to construct a better or more prolific breeding place.

The description is not overdrawn, and every physician should know that if this condition is changed the patient stands a better chance for recovery. The gauze method is dangerous, while the tooth brush is a necessity. The texture of the brush should be of the soft grade.

I wish to close this paper with a quotation from Dr. Talbot of Chicago giving his excellent prescription: "Patients present themselves with foeta of the breath, pus about the teeth, diseased gums, ropy saliva, with all forms of bacteria . . . . . which are taken into the stomach at every swallow and pass through into the intestines . . . . . In order to reach the deep-seated diseases of the gums iodine alone would penetrate the bone . . The official tincture of iodine contains 7% of iodine dissolved in alcohol, to which is added 5% of potassium iodide. This preparation if used will often cause the mucous membrane to become tender and raw, it will also, in some patients, destroy the mucous surface. To overcome this difficulty, many years ago I formulated the following, which I call Iodoglycerole:

| | |
|---|---|
| Water | 10 parts |
| Zinc Iodide | 15 parts |
| Iodine | 25 parts |
| Glycerine | 50 parts |

"As compared with the ordinary tincture of iodine its astringent properties are greatly increased, the glycerine causes a rapid absorption and the irritating effects are reduced to a minimum. The penetrating effect is remarkable—long, round wood applicators with cotton wound on one end are saturated with this preparation and the gum margin above and below painted. The lips and cheeks are held away from the jaws until the iodine has dried. Frequent applications will destroy all germs in the mouth and reduce mouth and general disease to a minimum.."

In conclusion—examine the teeth and gums with more care. Be careful of the "bumps and ulcers"—instruct patient in Oral Hygiene—and lastly use the Iodoglycerole treatment for all suspicious mouths, and I feel sure that the time you have spent in listening to the paper will not have been spent in vain.

319 Grant Building.

